# Real-world comparative effectiveness of analogue and human insulin on glycemic control in children and adolescents with Type 1 diabetes in Lebanon using continuous glucose monitoring data

**DOI:** 10.57264/cer-2026-0036

**Published:** 2026-07-16

**Authors:** Sahar Masri, Manuel Albela, Iza Ciglenecki, Philippa Boulle, Tania Hachem, Sola Bahous, David Prieto-Merino, Pablo Perel

**Affiliations:** 1 Médecins Sans Frontières, Beirut, Lebanon; 2 Médecins Sans Frontières, Geneva, Switzerland; 3 Médecins Sans Frontières, Amman, Jordan; 4 Lebanese American University, Beirut, Lebanon; 5 University of Alcalá, Madrid, Spain; 6 London School of Hygiene & Tropical Medicine, London, UK

**Keywords:** analogue insulin, continuous glucose monitoring, hypoglycemia, low-middle income countries, real-world data, time-in-range, Type 1 diabetes

## Abstract

**Aim:**

In high income settings, insulin analogues were associated with improved glycemic control among adults with diabetes, mainly measured through HbA1c and hypoglycemic episodes. In low- and middle-income countries, especially those affected by humanitarian crises, analogue insulins may be helpful as proper diabetes care for this population remains challenging; yet, supporting evidence is lacking.

**Materials & methods:**

Routinely collected continuous glucose sensor data from patients with Type 1 diabetes, aged 4 to 18 years old, between April 2019 and December 2022 was retrospectively extracted. Changes in glycemic metrics while using analogue insulins were compared with human insulin using multiple linear regression with random effects.

**Results:**

Forty-five patients included in the study used continuous glucose monitor devices for an average duration of 73 days (±53) while using human insulin and 170 days (±83) while using analogue insulins. Compared with human insulin, analogue insulins were associated with an additional 26 min (95% CI; 15, 37, p < 0.001) per day spent within the normal target range and a reduction of 11 min (95% CI; -16, -5) and 10 min (95% CI; -13, -7) in overall and nocturnal hypoglycemia, respectively. Analogue insulin was also associated with a reduction of 6 mg/dl in 24 h mean glucose.

**Conclusion:**

Insulin analogues were associated with slight improvements in time within and below range compared with human insulin, yet within patient glycemic variability remains high. Further research is needed to explore its acceptability among patients, impact on quality of life and its cost-effectiveness and sustainability in low resource contexts.

## Plain language summary: Effect of using analogue insulins compared with human insulin on management of Type 1 diabetes among children & adolescents in Lebanon

**What is this article about?:**

Patients living with Type 1 diabetes (T1D) are required to use synthetic insulin, a hormone used to manage their blood glucose levels. For many years, synthetic human insulin was the standard treatment, yet recent studies have shown that newer formulations of insulin, analogue insulins, result in better management of disease overall. These studies, however, have mainly been done in high income countries where analogue insulins are widely accessible. For patients in low-middle income countries, human insulin remains the standard treatment and access to analogues is limited.

This study aimed to provide evidence on the real-world effectiveness of these analogue insulins compared with human insulin among children and adolescents with T1D in Lebanon.

**What were the results?:**

Data used in this study was collected from continuous glucose monitoring devices that measure blood glucose levels over a 24 h period for the whole time the sensor is worn. Blood glucose levels were compared while using human insulin and analogue insulins over a long period of time.

Results showed that analogue insulin use was associated with improved diabetes management where patients had spent more time in the target range and less time in the dangerous lower ranges of blood glucose.

**Why is this important?:**

This study aimed to provide evidence from a population that has not been studied before. The researchers hope these results will help advocate for wider access to analogues at affordable prices for patients living with T1D in Lebanon.

The Middle East and North Africa region reported the highest age standardized diabetes prevalence in 2024, and in 2025 in Lebanon alone, 710 new cases of Type 1 diabetes (T1D) were estimated among children and adolescents under 20 years [1,2]. Reports from the International Diabetes Federation on the burden of diabetes in children and adolescents showed that low-and-middle income countries (LMICs) have disproportionately higher mortality rates compared with higher income countries (HICs) [3]. LMICs also accommodate 68% of refugee populations, and around 38% of forcibly displaced people globally are children younger than 18 years old [4]. However, epidemiological data on diabetic populations in crisis affected settings is very limited and available figures are not easily comparable given the heterogeneity of the reported disease classifications [5,6].

Analogue insulins have expanded treatment options to include those that more closely resemble the typical human physiologic insulin secretion and are now being classified as ultra long-, long- or rapid-acting insulin analogues depending on their onset and duration of action and peak time [7,8].

The HypoAna trial reported that treatment regimens including long- and rapid-acting insulin analogues were associated with a 6% reduction in the risk of severe hypoglycemic episodes compared with regular/NPH human insulin, and a 39% reduction of nocturnal hypoglycemic episodes among adult patients with T1D [9]. Other studies in HICs also reported that children and adolescents with T1D using long-acting analogue insulins had lower risks of hypoglycemia despite no changes in Hba1c levels [10,11].

In HICs, patients using analogues also reported greater treatment flexibility [12] and satisfaction [13] which may suggest better adherence compared with human insulin. However, due to significantly higher prices [6,14], supply challenges and health system fragility, their use in LMICs remains limited [15].

The limited studies on the feasibility and effectiveness of analogue insulins for people with T1D in LMICs, have mainly relied on assessing changes in Hba1c levels (considering Hba1c is the gold standard treatment end point) [16] or changes in hypoglycemic episodes through self-monitoring of blood glucose (SMBG) [17–19]. However, with the introduction of continuous glucose monitors (CGM), newly recognized glycemic metrics such as time-in-range and glycemic variability [16,20–22] have proven to be more insightful than static Hba1c values since they're able to show ‘real-time’ inferential data on glycemic control [16].

## Study aim

To our knowledge, real-world evidence on analogue insulin use in low resource contexts has been scarcely documented so far; so the aim of the study was to determine if using analogue insulin was associated with improved glycemic outcomes and variability among a pediatric cohort with T1D using real-world continuous glucose monitoring data in Lebanon.

## Materials & methods

### Study design & setting

This was an observational historical cohort study using previously collected routine clinical data between April 2019 and December 2022. No prospective interventions were done, however, for the analysis, analogue insulin use was treated as an exposure and the usual medication using human insulin was the comparator.

### Inclusion criteria

**Figure 1.**
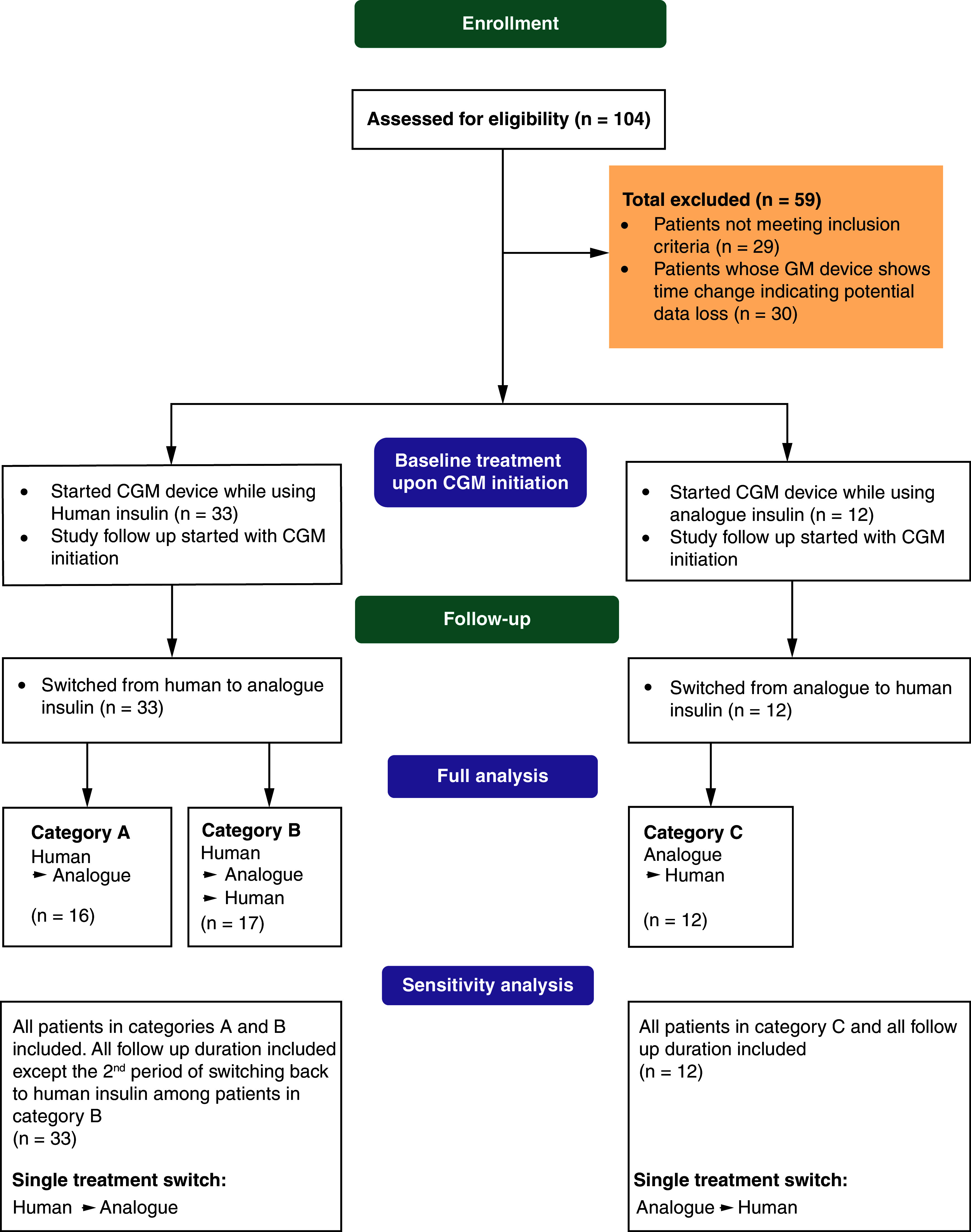
CONSORT patient flow diagram. CGM: Continuous glucose monitors.

To be included in the study, patients had to be between 4 and 18 years old; diagnosed with T1D; using CGM devices while using both types of insulin (analogue and human insulins) between April 2019 and December 2022 (Figure 1).

### Exclusion criteria

Patients who unknowingly changed the time settings on their CGM devices were excluded from the study because this could potentially overwrite the previously stored glucose readings, so previously recorded data would have been lost.

### Programmatic overview of T1D care in Médecins sans Frontières clinics in Lebanon

In Lebanon, Médecins sans Frontières (MSF) has been continuously providing free of charge noncommunicable disease care for Syrian refugees and the vulnerable host community in the North and Bekaa governorates since 2012. In April 2019, MSF piloted the use of CGM devices for pediatric patients with T1D to reduce the challenges of finger-prick testing for SMBG and facilitate more accurate insulin adjustments based on 24 h glucose readings. The CGM devices measure and record blood glucose every 15 min for a period of 14 days, after which the sensor should be replaced. After 14 days of use, the patients visit the clinic and the physician uploads the saved glucose data from the device to its cloud-based management system, Libreview. With each sensor change, the CGM data accumulate for each patient on the device’s software, and records of repeatedly measured glucose values would be available to download at any time. The expected number of glucose readings stored by the device are around 96 readings per day, if the user scanned the device every 8 h. For cost-constraints, MSF devices were only given once per month, and SMBG through finger prick testing was used for the remaining 14 days.

**Figure 2.**
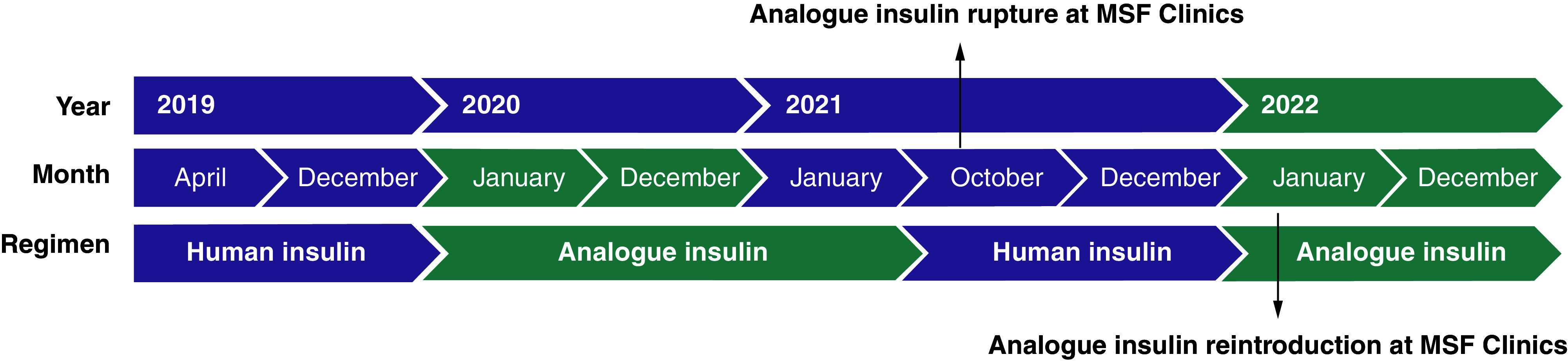
Treatment combinations during follow-up period. MSF: Médecins sans frontières.

As of January 2020, MSF introduced long- and rapid-acting insulin analogues (glargine 100 ui/ml and glulisine 100ui/ml) to pediatric patients with T1D as an alternative to the standard treatment (regular and NPH insulin). However, between October and December 2021, patients who were still part of the MSF cohort had to be switched back to the human insulin due to a country-wide shortage in analogues. Once the shortage was resolved, patients were switched back to analogues as of January 2022 (Figure 2). All human insulins were administered using vials and insulin syringes, and all analogue insulins were administered using insulin pens. All patients were on basal-bolus regimes.

Once the Covid-19 pandemic hit Lebanon as of the 1st of February 2020, infection prevention and control measures were consequently put in place in all MSF clinics. This included establishing pre- triage and triage pathways to identify and separate potentially suspected cases, use of face masks for all patients and implementing social distancing in the clinic waiting areas. However, these measures did not affect patients’ access to healthcare within the clinics, as follow-up consultations were still scheduled and patients continued to receive their treatments as the clinics remained open throughout the pandemic.

#### Sample size

Since the number of patients who used CGM devices in the MSF NCD cohort in Lebanon is limited, no sample size calculations were done, and all patients were assessed for eligibility.

### Data collection & analysis

#### Data sources

The main data sources were the individual patient glucose data collected from Libreview (linked to each patient’s CGM device) and patient records from MSF’s District Health Information System (DHIS2)

#### Data collection & cleaning

To obtain a complete dataset for analysis, rigorous data checking was done on CGM datasets extracted from Libreview (Supplementary Table 1). These datasets included only the recorded continuous glucose measurements and the date and time for each value (date-time dataset) per patient. More concise datasets were created where each of the glycemic metrics (described in the outcome section) was summarized for each patient on each recorded sensor date.

Other background variables such as age, sex, nationality, were matched for each patient ID from MSF’s patient records from DHIS2.

To be in line with the data standards recommended by International Consensus on Use of Continuous Glucose Monitoring [20,23], any day with less than 70% of expected CGM readings (96 expected readings per day) was discarded (Supplementary Table 1). Across all patients, 23.4% of all CGM days on human insulin were discarded and 20% of all CGM days on analogue insulin were discarded, so the ratio of CGM days on human to analogue insulin in the final dataset and original dataset were similar. Univariable and multivariable analysis were done using this complete dataset.

### Exposure variable

Analogue insulin was chosen as the exposure, and human insulin use was chosen as the comparator.

The type of insulin treatment (exposure variable) on each available date collected from MSF’s patient clinical records was coded as either analogue (identified as ‘1’) or human insulin (identified as ‘0’). The main exposure variable (type of insulin) would change throughout the follow-up period according to programmatic changes in MSF clinics throughout the follow-up period (Figure 2).

### Outcome variables

The main glycemic metrics per patient per CGM date used for analysis included:

- Total time spent in target range (70–180 mg/dl).
- Total time below range (TBR) (<70 mg/dl) and in severe hypoglycemia (<54 mg/dl).
- Nocturnal time below range (<70 mg/dl between 12:00 am and 6:00 am).
- Coefficient of variation of daily glucose values per patient (%glycemic variability).
- Mean daily glucose.

### Data analysis

Demographic and background information were presented as proportions or means and standard deviations. Univariable and multiple mixed effects model with the patient as random intercept to adjust for repeated outcome measurements were used to compare the average level for each glycemic metric when patients were on analogue insulin versus human insulin. Sex, age group at CGM initiation and the duration of treatment before the CGM introduction were adjusted for in the multivariable mixed models. Sensitivity analysis was done by exploring alternate statistical approaches to confirm direction of reported association between analogue insulin and the reported glycemic metrics and to control for temporal trends across the study follow-up duration.

All CIs were reported at the 95% level. We used R software for statistical analysis (version 4.3.2; R Foundation for Statistical Computing, Vienna, Austria) and the STROBE reporting checklist when editing the manuscript [24].

## Results

### Descriptive analysis

Forty-five pediatric patients diagnosed with T1D were included in the study, 53.3% were males and the majority (95.6%) were Syrian refugees. The mean age when the CGM device was introduced was 11.3 (SD 3.1) years. Fifty-three percent of the children had been treated at the MSF clinic for over a year before their first CGM use (Table 1).

**Table 1.** Demographic and baseline characteristics.

Patient characteristics	Overall (n = 45^†^)	Category A (n = 16^†^)	Category B (n = 17^†^)	Category C (n = 12^†^)	p-value^‡^
**Sex**					0.5
Male	24 (53%)	7 (44%)	9 (53%)	8 (67%)	
Female	21 (47%)	9 (56%)	8 (47%)	4 (33%)	
**Nationality**					0.3
Lebanese	1 (2.2%)	1 (6.3%)	0 (0%)	0 (0%)	
Syrian	43 (96%)	15 (94%)	17 (100%)	11 (92%)	
Palestinian	1 (2.2%)	0 (0%)	0 (0%)	1 (8.3%)	
**Age at CGM initiation**					0.034
12–18 years	22 (49%)	12 (75%)	6 (35%)	4 (33%)	
4–11 years	23 (51%)	4 (25%)	11 (65%)	8 (67%)	
**Treatment duration before CGM initiation**					0.012
more than 1 year	24 (53%)	11 (69%)	11 (65%)	2 (17%)	
up to 1 year	21 (47%)	5 (31%)	6 (35%)	10 (83%)	
**Treatment duration during study follow-up (days)**					
Human insulin	472 (193)	318 (195)	629 (45)	456 (147)	<0.001
Analogue Insulin	194 (128)	184 (101)	300 (86)	58 (59)	<0.001
**Overall CGM wear duration during study follow-up (days)**					
Human Insulin	73 (53)	54 (35)	125 (39)	27 (23)	<0.001
Analogue Insulin	170 (83)	115 (81)	238 (51)	148 (52)	<0.001

† n (%); mean (SD).

‡ Pearson’s Chi-squared test.

Category A: Human to analogue.

Category B: Human to analogue to human.

Category C: Analogue to human.

CGM: Continuous glucose monitors.

Between April 2019 and December 2022, patients used CGM devices for a mean duration of 73 days (SD 53) while using human insulin and 170 days (SD 83) while using analogue insulins. On average, 22.8 (SD 1.8) and 22.9 (SD 1.6) hours’ worth of glucose data were captured per day on human and analogue insulin, respectively (Table 1).

Overall, 983,001 glucose values (readings) were included in the analysis; 295,561 (30%) readings captured while using human insulin and 687,440 readings (70%) while using analogue insulins (Supplementary Table 2). There were no significant differences in the reported patient demographic information between those who started on human insulin then switched to analogue and those who started on analogue and then later switched to human (Table 1).

### Glycemic metrics analysis

While using human insulin, time in rage (TIR) was on average 378 min (95% CI; 343; 412) and increased to 403 min (95% CI; 370, 437) with analogue insulin. TBR was on average 88 min (95% CI; 76, 101) with human insulin and was reduced to 78 min (95% CI; 66, 90) with analogue insulin. Analogue insulin was also associated with a 10-minute (95% CI; -13, -7) reduction in nocturnal TBR. Results of the univariable and multivariable analyses are shown in Table 2.

**Table 2.** Crude and adjusted effects of analogue insulin on glycemic metrics.

	Crude estimates (95%CI)	p-value^†^	Adjusted estimates^‡^ (95% CI)	p-value^†^
**Time in range (70–180 mg/dl) (minutes)**				
Human insulin (reference)	378 (343, 412)	<0.001	376 (341, 412)	<0.001
Analogue insulin	403 (370, 437)		402 (368, 437)	
Effect estimate (difference)	26 (15, 37)		26 (15, 37)	
**Time below range (<70 mg/dl) (minutes)**				
Human insulin (reference)	89 (76–101)	<0.001	88 (76, 101)	<0.001
Analogue insulin	78 (66, 90)		78 (65, 90)	
Effect estimate (difference)	-11 (-16, -5)		-11 (-16, -5)	
**Time in severe hypoglycemia (<54 mg/dl) (minutes)**				
Human insulin (reference)	41 (33, 49)	<0.001	41 (33, 50)	<0.001
Analogue insulin	34 (26, 41)		34 (26, 42)	
Effect estimate (difference)	-8 (-11, -4)		-8 (-11, -4)	
**Nocturnal time below range (<70 mg/dl) (minutes)**				
Human insulin (reference)	35 (30, 40)	<0.001	35 (30, 40)	<0.001
Analogue insulin	25 (20, 29)		25 (20, 29)	
Effect estimate (change)	-10 (-13, -7)		-10 (-13, -7)	
**Glycemic variability (coefficient of variation), %**				
Human insulin (reference)	41 (39, 42)	0.11	41 (39, 42)	0.11
Analogue insulin	40 (39, 42)		40 (39, 42)	
Effect estimate (difference)	-0.4 (-1, 0.1)		-0.4 (-1, 0.1)	
**24 h mean glucose (mg/dl)**				
Human insulin (reference)	248 (237, 259)	<0.001	248 (237, 259)	<0.001
Analogue insulin	242 (232, 253)		242 (231, 253)	
Effect estimate (difference)	-6 (-8, -3)		-6 (-8, -3)	

Crude and adjusted effects of using analogue insulin (glargine/glulisine) on reported glycemic metrics compared with human insulin (regular/NPH) (N = 45 patients; 10,967 days; 983,001 glucose readings).

† *t*-tests using Satterthwaite’s method.

‡ Adjusted for sex, age group at CGM start, treatment period before CGM start.

The daily mean glucose while using human insulin was estimated at 248 mg/dl (95% CI; 237, 259) and it was reduced to 242 mg/dl (95% CI; 232, 253) with the use of analogues.

However, we did not find evidence of reductions in the percentage of within-day glycemic variability (% coefficient of variation of 24 h glucose per patient).

These reported comparisons between treatment groups did not show any substantial changes after adjusting for sex, age group at CGM initiation or the duration of treatment before the introduction of CGM devices (Table 2).

**Figure 3.**
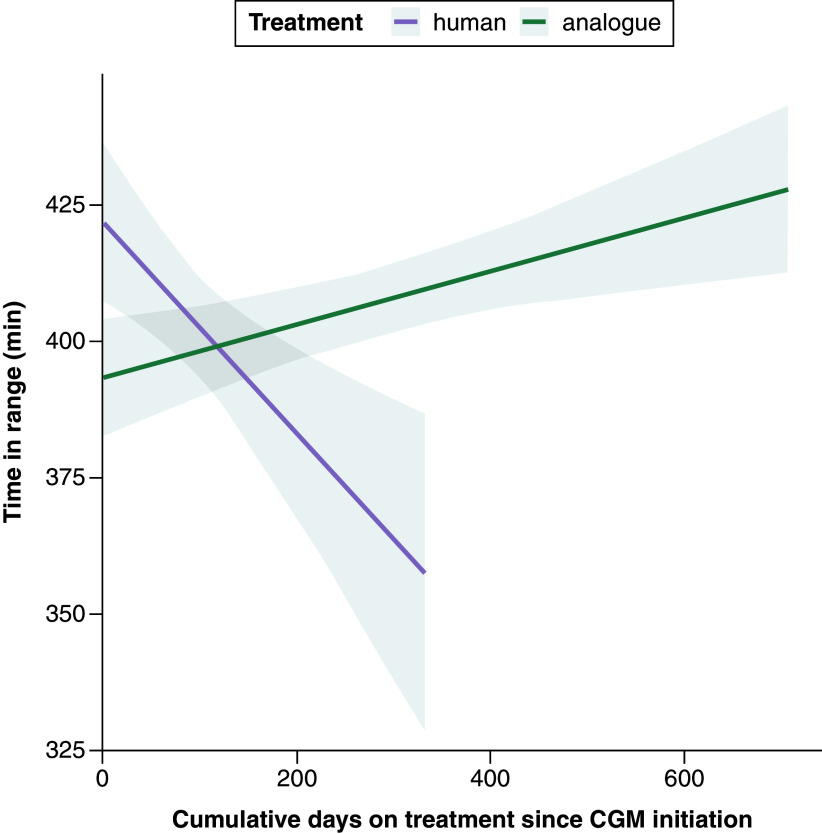
Predicted linear trends of time in range per treatment type. CGM: Continuous glucose monitors.

Figure 3 shows the predicted trends of TIR for analogue insulin compared with human insulin per cumulative days of treatment.

## Sensitivity analysis

A random effects model adjusting for patient categories based on the type of treatment switch throughout the follow-up period (human to analogue) versus (analogue to human) showed similar effect estimates for analogues regardless of the treatment switch (Supplementary Table 3). Further analysis of TIR trends also showed that while the TIR on human insulin declined, analogue insulins showed a more stable trajectory throughout the follow-up period Supplementary Figure 1).

To control for shared temporal trends and time-varying confounding, a fixed effects model also reported favorable results for analogue insulin compared with human insulin regarding TIR and mean daily glucose (Supplementary Table 3).

## Discussion

To our knowledge, this is the first study to assess the effect of analogue insulins on glycemic control among pediatric patients with T1D in a LMIC affected by humanitarian crises all while using continuous glucose monitoring data.

One critical study finding was that analogue insulin was associated with a 12% relative reduction in TBR (<70 mg/dl) and a 28% relative reduction in nocturnal TBR. Given the high prevalence of impaired awareness of hypoglycemia among children with T1D [25] and the critical impact of severe hypoglycemic episodes on cognitive impairment [26], analogue insulins may offer improved glycemic outcomes with lower risk of developing hypoglycemia as treatment duration progresses.

Another important outcome of this study was that analogue insulin was associated with a 6.8% relative increase in TIR. Although from an individual clinical perspective, the absolute effect (measured in minutes/day) on glycemic metrics might seem small, its long-term impact on health outcomes cannot be understated, especially for children with T1D [26]. A widely referenced study by Beck *et al.* reported that a 10% decline in TIR increased the hazard ratio for developing retinopathy by 64% and that a difference of only 2.5 h per day in TIR was enough to distinguish between those who developed a microvascular outcome and those who didn't [21]. This association was also evident among patients with T1D in another CGM study [27]. The same change in TIR, was also correlated with a 0.5–0.6% reduction in Hba1c [20,28].

Despite such improvement, the overall TIR remained sub-optimal as it falls around 8 h short of the internationally set targets (recommended at 14 h a day) [20,21], although more realistic targets are recently being suggested for children [29]. However, for children living in settings with limited resources, such glycemic targets are often unachievable due to the plethora of documented challenges [5,14,15]. This sub-optimal glycemic control, regardless of the treatment provided, is consistent with that seen in similar populations with poor socioeconomic and living conditions; where the majority of underprivileged patients are at higher risk of poor glycemic control and more prone to have hypoglycemic episodes compared with others [17–19].

Lastly, the estimated level of within-day glycemic variability, an indicative marker of daily glycemic excursions, remained stable at around 40%. This exceeds the recommended cut-off level for glycemic variability (36%) distinguishing stable from unstable patients with diabetes [30]. The level of glycemic variability shown here, however, is similar to that reported in another study on patients with T1D [31] indicating potentially poorer glycemic control among children with T1D in general. This is worth noting, especially since reducing glycemic variability is often seen as a potential risk for hypoglycemia, which was not evident here. In our study, the differences in 24 h mean glucose and glycemic variability between human and analogue insulins were both very minimal, perhaps because the time spent above range had increased balancing out the increase in time spent in range to arrive at the same 24 h mean glucose. The consistent level of glycemic variability across both treatments suggests that extreme glucose excursions far into the hyperglycemic range persisted with analogue insulins despite the reduction in hypoglycemic readings.

As this study is meant to reflect real-world application of analogue insulins for children with T1D, it should be emphasized that while participants in this study had access to insulin free-of-charge, many children with T1D in low-middle income settings still face challenges in accessing such treatments due to their high cost compared with standard human insulin. A recent cost of production study by MSF showed that a treatment regimen using analogue insulins in a reusable pen device could only cost USD 111 per year, which is two-times cheaper than the lower range of the current market price, ranging from USD 312 to USD 690 per year. This further emphasizes the need to advocate for wider use of generics and biosimilars and a more competitive market rather than the available oligopoly of manufacturers [32].

## Limitations

The relatively newer glycemic metrics used in this study have only recently gained attention as validated research outcomes [21] which explains the limited studies using these metrics to compare human to analogue insulins, especially those including children and adolescents with T1D.

One major limitation, inherent to CGM implementation within MSF clinics, was that the devices were generally only worn for 14 days each month, meaning there were gaps within the overall follow-up period for each patient. Therefore, we could not account for potential changes on the days when the sensors were not worn. Another limitation is potential selection bias given the number of patients excluded because they changed the time settings on their devices (n = 30). Since we found no significant differences in the background characteristics between those included in the study and those excluded for changing the device's time settings, we believe this bias might have impacted the observed association in either direction.

The sample size available was relatively small and chance could have affected the precision of the reported estimates. A further concern is that insulin dosage adjustment and the extent of its shift between analogue and human insulin is not reported here, which might have been a potential confounder. We also believe that given analogue insulins were administered through insulin pens, this might have confounded the observed association especially that use of insulin pens alone may lead to more favorable results compared with vials.

Given the limited resource setting and context of the study, the findings might not be generalizable to all children and adolescents living with T1D. We anticipate however, they might be more relevant for healthcare workers and patients in LMICs where access to insulin analogues is limited.

The random effects model used in this study, while it accounts for time-invariant patient differences, it may not be suitable to account for time-varying potential confounders such as changes in diet, physical activity, disease progression, puberty, psychological stress, or CGM learning effects. The effect of these potential confounders on glycemic control throughout the study duration which also took place during the COVID-19 pandemic remain unaddressed and we therefore consider this a limitation of the current analysis, even though the results of the sensitivity analysis supported the reported findings.

## Strengths

The main strength of this study is that it is the first to compare analogue to human insulin among pediatric patients in a LMIC affected by humanitarian crises using CGM data.

Using CGM data enabled a detailed analysis of the level of glycemic control among this vulnerable population, which had not been previously documented. Compared with other studies, hypoglycemic outcomes were ascertained through the CGM method, thereby ensuring no hypoglycemic events were overlooked or undocumented as in SMBG. Despite the limited sample size, a large number of glucose readings were analyzed.

The methodology and statistical analysis using mixed effects models are also a key strength as many studies have been mainly descriptive. We believe residual confounding by time-invariant patient characteristics is unlikely, since using a random effects model controls for potential effects of unmeasured baseline patient characteristics on the reported outcomes. So essentially each patient acts a self-control. Sensitivity analysis reported comparable favorable results for analogue insulins regarding improved TIR and 24 h mean glucose levels.

The relatively long follow-up duration before and after introducing analogues might have also controlled for any impact on glycemic control introduced by initial use of CGM devices.

## Conclusion

Overall, the study showed that the use of analogue insulins was associated with improved glycemic control. Despite no changes in glycemic variability, analogues were associated with a reduction in time-below-range and an increase in time-in-range. The small but positive impact seen at the individual level, may translate to improved blood glucose levels over longer periods of time and more favorable health outcomes later in life, if analogues were more accessible and affordable. This may also positively impact the quality of life for those living with T1D in unstable conditions or with limited access to resources. Future qualitative studies are strongly encouraged to explore analogue insulins’ impact on quality of life or treatment adherence and satisfaction among this population.

## Summary points

- Studies have shown that analogue insulins result in improved glycemic control; however, evidence from the pediatric population in low-and-middle income countries remains very limited.
- This study used real-world data collected from continuous glucose monitoring devices worn by 45 children and adolescents living with Type 1 diabetes attending primary healthcare clinics in Lebanon.
- Patients used sensor devices for a mean duration of 73 days while using human insulin and 170 days while using analogue insulins.
- While using analogue insulins, patients spent on average an additional 26 min per day in the recommended target range.
- The time spent in overall and nocturnal hypoglycemia was also reduced by 11 min (95% CI; -16, -5) and 10 min (95% CI; -13, -7), respectively, while using analogue insulins.
- The daily mean glucose while using human insulin was estimated at 248 mg/dl and it was reduced to 242 mg/dl with the use of analogue insulins.
- There was no evidence of changes in the percentage of within-day glycemic variability.
- Despite slight improvements seen with analogue insulin use, the additional time spent in target range among youth living with diabetes might have a positive impact on long term health outcomes.

## Supplementary Material


