# Supplementary material for: Real-world comparative effectiveness of analogue and human insulin on glycemic control in children and adolescents with Type 1 diabetes in Lebanon using continuous glucose monitoring data
Source: J Comp Eff Res. 2026 Jul 16;15(8):e260036. doi: 10.57264/cer-2026-0036 (PMC13436064; doi:10.57264/cer-2026-0036)
Supplement: Supplementary file 1 [file cer-15-260036-s1.docx]

**Supplementary Materials**

**Supplementary Figure 1. Predicted trends of time-in-range stratified by treatment switch.**

| **Supplementary Table 1. Different versions of datasets.** | | | |
| --- | --- | --- | --- |
|  | **Date-Time dataset*** | **Date Only dataset*** | **Valid dates dataset** |
| Total patients included | 45 | 45 | 45 |
| Total CGM Days | 13 763 | 13 763 | 10 967 |
| Total days on human | 4 310 (36%) | 4 310 (36%) | 3 301 (30%) |
| Total days on analogue | 9 453 (64%) | 9 453 (64%) | 7 666 (70%) |
| Rows in panel dataset | 1 101 246 | 13 763 | 10 967 |
| *Date Only dataset is a concise version of Date-Time dataset. It includes only dates rather than date and time, so it has the same information and the same number of patients and glucose readings. | | | |
|  | | | |

| **Supplementary Table 2. Total glucose readings per treatment.** | | | | |
| --- | --- | --- | --- | --- |
|  | **CGM Days** | **Glucose readings** | **Total hours captured** | **Mean hours per day** |
| Human insulin | 3301 | 295561 | 75169 | 22.8 (± 1.8) |
| Analogue insulin | 7666 | 687440 | 175312 | 22.9 (± 1.6) |

| **Supplementary Table 3. Sensitivity analysis.** | | | | |
| --- | --- | --- | --- | --- |
|  | **Full reported model**  (All patients and switching episodes)  N= 45 patients  (10967 days) | **Alternative Model 1**  Adjusted for switch category  (All switching episodes)  N= 45 patients  (10967 days) | **Alternative Model 2**  Adjusted for switch category  (One switch episode)  N= 45 patients  (10119 days) | **Alternative Model 3**  Adjusted for calendar month  (One switch episode)  N= 45 patients  (10119 days) |
|  | Mixed model with patient random intercept | Mixed model with patient random intercept | Mixed model with patient random intercept | Fixed effect model |
| **TIR (minutes)**  Effect estimate  [95%CI] | 26  [15 – 37]  (p-value <0.001) | 26  [15 – 37]  (p-value <0.001) | 25  [13 – 37]  (p-value <0.001) | 102  [44 – 159]  (p-value <0.001) |
| **TBR (minutes)**  Effect estimate  [95%CI] | -11  [(-16) – (-5)]  (p-value <0.001) | -11  [(-16) – (-5)]  (p-value <0.001) | -13  [(-19) – (-6)]  (p-value <0.001) | 17  [(-11) – 44]  (p-value: 0.2) |
| **24-hr Mean Glucose (mg/dl)**  Effect estimate  [95%CI] | -6  [(-8) – (-3)]  (p-value <0.001) | -6  [(-9) – (-3)]  (p-value <0.001) | -4  [(-7) – (-1)]  (p-value <0.01) | -34  [(-54) – (-15)]  (p-value <0.01) |
| Sensitivity analysis for the association between analogue insulin and the times within and below target range and mean daily glucose.  TBR: Time below target range; TIR: Time in target range. | | | | |
